# Phages for Phage Therapy: Isolation, Characterization, and Host Range Breadth

**DOI:** 10.3390/ph12010035

**Published:** 2019-03-11

**Authors:** Paul Hyman

**Affiliations:** Department of Biology/Toxicology, Ashland University, 401 College Ave., Ashland, OH 44805, USA; phyman@ashland.edu; Tel.: +1-419-207-6309

**Keywords:** phage therapy, bacteriophage isolation, host range, bacteriophage characterization, genome sequencing, enrichment culture

## Abstract

For a bacteriophage to be useful for phage therapy it must be both isolated from the environment and shown to have certain characteristics beyond just killing strains of the target bacterial pathogen. These include desirable characteristics such as a relatively broad host range and a lack of other characteristics such as carrying toxin genes and the ability to form a lysogen. While phages are commonly isolated first and subsequently characterized, it is possible to alter isolation procedures to bias the isolation toward phages with desirable characteristics. Some of these variations are regularly used by some groups while others have only been shown in a few publications. In this review I will describe (1) isolation procedures and variations that are designed to isolate phages with broader host ranges, (2) characterization procedures used to show that a phage may have utility in phage therapy, including some of the limits of such characterization, and (3) results of a survey and discussion with phage researchers in industry and academia on the practice of characterization of phages.

## 1. Introduction

The isolation of bacteriophages for phage therapy is often presented as a fairly straightforward exercise of mixing a phage-containing sample with host bacteria, followed by a simple removal of bacterial debris by filtration and/or centrifugation the next day [1,2,3]. Indeed, when questions of bacterial resistance to therapeutic phages are discussed, it is common to hear some variation of “in that case, we will just isolate another phage” [4]. Beyond the implication that there is a very large, if not infinite supply of different phages for a target host and that there is no time or other cost involved in isolating and characterizing a novel phage, this idea also presumes that all lytic phages will be equally effective for phage therapy. (Note that unless explicitly stated, I will use “lytic phage” to refer to obligately lytic phages and “temperate phage” to refer to phages that can replicate using lytic or lysogenic life cycles.) In actuality, lytic phages vary in many properties such as length of infection, number of progeny produced and of especial importance to phage therapy, host range.

Host range has several effects on the utility of a phage for use in phage therapy. A host range limited to a single species is desirable as it prevents the phage from killing other species, leaving the rest of the host’s microbiome intact. So in terms of bacterial species, the phage should have a narrower host range. Within that bacterial species, however, a phage that infects many, if not all strains is useful because it means that many bacterial infections by that species can be treated somewhat empirically (i.e., presumptively), that is, without having to test the sensitivity of the infecting strain for susceptibility. This is analogous to the use of broad-spectrum antibiotics (antibiotics that affect bacteria across genera, family, order or even broader taxonomic ranges) before pathogen identification or antibiotic sensitivity testing [5]. For phage therapy, use of broader host range phages would presumably lead to fewer treatment failures due to a mismatched host and phage combination. So in terms of strains within target species, a broader host range is desirable. (Note that I will subsequently limit the use of the term “broad host range” because this term has been overused and misused within the phage literature to the point of no longer being useful. See Ross et al. [6] for discussion of this. This does not preclude the use of “narrower” and “broader” to compare host ranges.) One important caveat, which I will discuss further, is that host range testing under laboratory conditions may not completely reflect effective host ranges as observed in the patient. Nevertheless, as stressed here, it is certainly desirable to at least strive for desirable host range breadth earlier rather than later during preclinical development of phages for phage therapy.

Implicit in the previous paragraph is that an infection to be treated by phage therapy is an infection by a single species of bacteria. This is only sometimes true. Some infections are mixed infections (polymicrobial). This is often addressed through the use of mixtures of therapeutic phages (cocktails). I will discuss phage cocktails later in this article.

In this article I first review methods of phage isolation including some variations on the more common methods. Several of these variations were developed to specifically isolate phages with broader host ranges. I then discuss the characterization of newly isolated bacteriophages in general, and especially with respect to host range. Finally, I present the results of interviews and surveys of phage workers from both academic and industrial labs with a focus on the standards of phage characterization, again, especially host range testing.

## 2. Phage Isolation Methods

A bacteriophage’s host range can determine the phage’s utility for phage therapy. Some phages will infect only a few strains (narrower host range), others will infect many strains of the same species (broader host range), and a few phages can infect more than one species (polyvalent host range). Standard isolation protocols do not seem to select for any particular host range. Host range, instead, is usually characterized after the phage is isolated, with isolation typically accomplished on a single strain of bacteria [2,3,7], although some groups favor using a mix of multiple host strains for phage isolation [8,9,10]. In this section, I consider phage isolation especially from a perspective of better tailoring resulting phage host ranges towards intended uses.

### 2.1. Overview of Isolation Protocols

The basic method of phage isolation has remained unchanged since it was developed by Felix d’Herelle. This is usually described as the enrichment procedure [3,8]. A sample of bacteria is mixed with an environmental sample and the mixture incubated for a time, typically overnight. The remaining bacteria are then removed from the culture by centrifugation and/or filtration and the filtrate is assayed for the presence of phages. Any phages identified are then characterized to determine whether they have the desired properties for phage therapy (as delineated in Lobocka et al. [8] and Abedon et al. [11], for example). Their usefulness may depend on phage virulence, host range, obligately lytic vs. other life cycles and so on. In general, it is difficult to screen for many of these properties during isolation, so they are tested for afterwards although there are exceptions. For example, filamentous phages tend to chronically infect hosts but can be excluded from isolation by including chloroform treatment in the isolation as chloroform tends to inactivate both filamentous and lipid containing bacteriophages [12].

Some researchers are able to isolate phages without doing enrichment cultures by plating environmental samples directly with an isolation host and looking for spot clearing or plaque formation [13,14]. This requires a relatively higher concentration of phages in the sample than for enrichment as only a limited volume of environmental sample can be used on a plate (tens of microliters for spot testing, a few milliliters for plaquing; see Table 1 in Section 3 below for details of these detection methods). Direct plating nevertheless has been used to isolate novel phage against *Escherichia coli* from stool samples [15] and from sewage effluent [16], phages from saliva that infect one of several species of oral bacteria [17], and phages from dental plaques (i.e., biofilm) that also infect oral bacteria [18]. Direct plating does have the advantage that any biases introduced by sample processing can be avoided. Plating conditions can also be varied to isolate bacteriophages that do not grow well under standard conditions. For example, Serwer and colleagues [19] used low density agarose media to isolate bacteriophages that did not plaque well because of their large size or forming aggregates, both of which appear to limit diffusion in standard media. The presence of the gel fibers may also promote the growth of some phages that do not lyse cells in broth culture [20,21]. This technique is very useful in finding phages in environmental studies where a broad diversity of isolated phages better reflects the viral community [20,22] but is not in widespread use isolating phages for phage therapy. For further discussion of these isolation methods and their potential advantages in phage therapy see [21,23].

Because many samples do not have high concentrations of phages for the desired host species needed for direct plating, most phages are isolated using an enrichment protocol. The major variations in this basic method are seen in: (i) the type of environmental sample, (ii) the type of pre-infection processing, (iii) the choice of isolation host(s) and how they are combined with the processed environmental sample, and (iv) post-infection processing and detection of the phage.

### 2.2. Environmental Samples

A general rule of thumb for finding bacteriophages is to look where the host is. Thus phages of photosynthetic planktonic bacteria can be isolated from photic seawater [24,25], phages for farmed fish pathogens have been isolated from coastal waters or fish farm water [26,27], phages for mammalian intestinal bacteria are readily isolated from fecal material, sewage, and farm run-off [28,29,30,31,32], while phages for bacteria causing human skin infections can be found on skin [33,34], healthy throat secretions [35], or in wound exudates [36], and so forth. For phage therapy, when a phage against a particular pathogen is desired, the source sample can be more specific such as looking at sewage lines from sewers leading from a hospital [37] or in patients themselves [36,38].

Despite this apparent ease at finding phages in many environments, finding a phage against a particular host may not be as easy. Mattila and colleagues [39] evaluated the likelihood of finding phages that infect particular antibiotic resistant bacteria using sewage as the source. While they were able to easily find phages infecting antibiotic resistant *Pseudomonas aeruginosa*, *E. coli*, *Salmonella*, and *Klebsiella pneumoniae*, phages infecting vancomycin resistant *Enterococcus* (VRE) and *Acinetobacter baumannii* were found in less than half the samples and phages infecting methicillin resistant *Staphylococcus aureus* (MRSA) were very infrequently isolated. In general however, it seems that a phage for at least some strains of every bacterial species can be found if enough environmental samples are tested. This may not be true for all combination of bacteria and phage types, however. Notably, all known *Clostridium difficile* phages are temperate phages isolated by induction of prophages [40].

### 2.3. Sample Processing

The goal of post-collection, pre-infection processing is to obtain phages at a sufficient titer in appropriate media for efficient infection of the isolation host bacteria. Different types of samples usually require specific types of processing. For example, virus titers, including phage titers in sea water can be relatively low necessitating preparatory concentration of the sample [41] that is typically done by filtration, precipitation, or a combination of both [3,42]. Czajkowski and colleagues [43], for example, demonstrated that zinc chloride could be used to concentrate phages from water or plant or soil extracts sufficiently to detect phages by direct plating without enrichment culture. They suggested that this gave a more accurate sampling of the diversity of phages in their samples. Bacteriophages in dilute samples can also be concentrated by flocculation (phages form small insoluble aggregates (flocs) that drop out of suspension even when the phage concentration is low) instead of precipitation (phages form a large insoluble mass (precipitant) [44,45]. In another interesting variation, phages were concentrated by adherence to and washing from bituminous coal [46]. Dry soil generally requires extensive washing to release phages that may be adhering to soil particles [47,48] although this washing may be bypassed [20]. Sewage samples often need the least processing, with just low-speed centrifugation used to remove solid particles [37,49]. Filtration is less common for these samples because the filters quickly become clogged.

Most workers end the processing of all samples with a 0.45 or 0.22 µ filtration step to remove endogenous bacteria, with the filter size choice depending on whether they are more concerned with removing all bacteria but also the largest bacteriophages (0.22 µ) or retaining very large bacteriophages at the expense of some bacterial carry through (0.45 µ). This step is omitted in some of the direct plating methods discussed in Section 2.1 above. In my lab, we have found that we sometimes get better results by omitting this filtration step when isolating phages from fecal samples and soil. Solids and bacteria are instead removed by centrifugation and filtration after the initial rounds of infection (P. Hyman, unpublished results). Rombouts and colleagues also used this variation to isolate phages against *Pseudomonas syringae* pv.*porri* [50]. My speculation is that directly mixing the sample and bacteria allows phages that are still adhering to particles to infect cells as long as the phages’ receptor binding proteins remain exposed. Several groups have shown that bacteriophages can infect bacteria even when the head is attached to a solid surface [51,52,53]. It seems a reasonable assumption that this is true of many if not all tailed phages. As well, phage growth during the initial enrichment step may be enhanced by the presence of additional host bacteria in the environmental sample. This presumes that phages and some susceptible hosts will be found in the same sample.

### 2.4. Choice of Isolation Host(s) and Enrichment Culture Variations

Choice of isolation host is perhaps the most critical part of the isolation process because the isolation host will constrain the types of phages isolated, particularly in terms of their host range. This is in some ways obvious—isolating phages using a Gram-negative host strain, for example, is unlikely to yield phages that infect a Gram-positive organism. However, as discussed below in the context of host range, it is not always true that newly isolated phages will only infect the isolation strain or species. It is more accurate to say that in general newly isolated phages will be constrained to those able to infect a host that displays the same general type of receptors as the isolation host, although again there are exceptions such as coliphages P1 and Mu, both of which have two sets of tail fibers for infecting two groups of hosts [54].

In some cases though, receptor specificity can be exploited to enhance the efficacy of phage therapy. For example, Filippov and colleagues isolated phages that used the lipopolysaccharide (LPS) of *Yersinia pestis* as a receptor [55]. Phage resistant strains of *Y. pestis* were less virulent when their phage resistance was due to an altered or partly deleted LPS caused by mutation of different LPS synthesis genes. Similarly, Lin and colleagues isolated *Klebsiella pneumoniae* phages that used the K1 capsule (a *K. pneumoniae* virulence factor) as their receptor [56]. Capsule-less strains were phage resistant but less virulent. A third example is phage OMKO1 isolated by Chan and colleagues which infects multi-drug resistant strains of *P. aeruginosa* using a component of the drug resistance efflux pump as the phage’s receptor [57]. Phage resistant mutants were more sensitive to antibiotic killing as the same mutations that prevented phage binding also reduced or eliminated the receptor protein’s activity as a component of the drug efflux pump.

Practical considerations can play a role in isolation-host choice—some bacterial species are difficult to culture in the laboratory or may be too pathogenic for easy use. Consequently, strains that are more amenable to routine culture may be used instead. For example, phages against mycobacteria are commonly isolated against *Mycobacterium smegmatis*, a relatively non-pathogenic species that can produce a bacterial lawn in a few days compared to weeks or months for *Mycobacterium tuberculosis* [58]. Of course, when a surrogate host is used, later host range testing against pathogenic strains is essential.

If clinically or environmentally isolated bacteria—in contrast to established, characterized laboratory strains—are to be used for phage isolation, it is wise to test the isolation strains for the presence of inducible prophages [59]. This can be done by exposing the bacteria to a DNA damaging agent such as UV light or mitomycin C [60,61] and plating to look for plaques or by sequencing the bacterial genome and looking for prophage sequences. For this latter approach multiple software tools have been developed specifically to identify prophages [62,63,64]. As a general principle, temperate phages are usually considered undesirable for phage therapy (see Section 3.4 below as well as Gill and Hyman [7] and Kropinski [65]). Thus, it is important to not use isolation hosts that might be induced to release temperate phages as enrichment strains.

Ghugare and colleagues recently published an alternative enrichment culture method that might be useful when phage concentrations in a processed sample are very low [66]. They immobilized the isolation host bacteria on a 0.45 μ filter and then immediately passed a processed water sample (150–300 mL) through the filter. The filter was then placed in nutrient media for incubation and phages recovered from culture at a higher rate than if the water sample and bacteria were mixed in a more standard enrichment culture they performed. It is important to note that while the filter would capture bacteria, it would not stop bacteriophages so the increase in efficiency appears to be from a combination of exposing the entire sample to bacteria and, perhaps, that the bacteria were effectively at a high concentration on the filter, acting as a trap for any phages able to bind to the bacteria. In separate experiments they tested this method with newly isolated strains of *Escherichia*, *Enterobacter*, *Salmonella*, *Shigella*, *Pseudomonas*, and *Vibrio*.

While most phages have been isolated using a single host strain, some interesting variations using multiple hosts have been developed. The first variation is to use more than one host strain during the enrichment protocol, usually multiple strains of the same species. Although there has been no direct comparison of phages isolated on one vs. several host strains there are indications that use of multiple strains may improve isolation of broader host range phages. This method has been used to isolate phages against *Clostridium sporogenes* [45] *Enterococcus faecalis* [6], *E. coli* [10], and *Salmonella* Enteritidis [67], among others. Ross and colleagues did find an increase in host range in two phages isolated on two strains of *E. faecalis* compared to two phages separately isolated on one strain but the small numbers of phages that were each isolated from independent environmental samples make this suggestive rather than conclusive [6]. A second reason for using multiple strains of the same species is to increase the likelihood of isolating a desirable phage from any individual sample by providing multiple host strains (J. Azeredo, personal communication). It is important when using this method to test isolation strains for antagonism so that all will grow equally well during enrichment. If environmental or clinical isolates are included in the hosts, they should also be screened for lysogeny as previously mentioned [67].

A second variation is to use several different species of bacteria during the enrichment culture. This method was first shown by Jensen and colleagues who used *Spheroplastin natans, E. coli* and *P. aeruginosa* to isolate polyvalent bacteriophages [32]. They did this with different pairs of strains as well as all three together. Interestingly, after isolation they found that propagating the phages on one host led to a reduction in efficiency of plating on the other isolation host but the few phages produced by culture on the second host then showed efficient growth on the second host but poor growth on the original propagation host. Poor growth on a host followed by good growth by the few surviving phages is reminiscent of classical restriction (due to restriction endonucleases). This pattern suggests that in addition to being able infect multiple species of bacteria, the phages had to escape some resistance mechanism when switching host species.

Another variation was described more recently by Yu and colleagues [68]. They developed this method after failing to obtain polyvalent phages using Jensen’s method. In this variation, they used two methods of phage cocktail growth on multiple hosts sequentially, that is, one host at a time. In the end, phages were isolated from a remaining mixture of phages that had been grown on every host. The first way this was done was by taking a filtrate of phages separated from a processed sewage sample (incubation in buffer to free phages from particles, followed by filtration to separate phages from other components) and applying the filtrate to a bacterial lawn. All plaques were harvested and grown in broth culture with a second host. This phage culture was then plated with the second host and all the resulting plaques were collected and used to create a new phage cocktail that was cultured on a third host and so on up to five hosts. In the second method of sequential host growth, the phage cocktail was mixed with a host. Cells including infected cells were then collected by centrifugation, leaving unadsorbed phage in the supernatant. The infected cells were then incubated to produce the next round of phages. For both methods they selected bacteria from among two strains of *E. coli*, two strains *P. aeruginosa*, one strain of *Pseudomonas syringae* and one strain of *Pseudomonas putida* in various combinations. At the completion of all rounds of phage growth, individual plaques were harvested to isolate new phages. Testing showed that the final isolated phages could infect all hosts in the isolation protocol. While the efficiency of plating was not identical between the hosts, it was with one exception between 0.45–1.15 compared to the propagation host. This is in contrast to the results of Jensen and colleagues [32], outlined above, where the efficiency of plating could vary by as much as 10^7^-fold between hosts.

Mapes and colleagues [69] also demonstrated a method to isolate phages with improved host range starting with a mixture of previously isolated phages. While this procedure starts with characterized phages rather than an environmental sample, it demonstrates another way culturing methods can be used to modify host range. Their host range expansion (HRE) protocol began with the mixing of an increasingly dilute cocktail of four phages with a series of *P. aeruginosa* strains in parallel. Phages from the most dilute mixture which lysed that host were collected and pooled. The pooled phages were then used to inoculate fresh hosts for the next round of HRE. After 30 cycles, the evolved phage cocktail, and individual phage clones isolated from the cocktail, had broader host ranges than the original four starter phages.

Except for the more complex procedures of Yu et al. [68] and Mapes et al. [69], once one or more enrichment strains have been chosen the typical enrichment culture is accomplished by mixing freshly grown bacteria with part of the processed environmental sample in fresh media. This mixture is then incubated under conditions appropriate to the host. After some hours or days, as appropriate for the growth conditions and enrichment host or hosts, the putative phage culture is processed as described below.

### 2.5. A Further Note about Hosts

The above discussion is directed at host choices and their uses in phage isolation. Even when isolation is successful, the host(s) used may not be optimal for other purposes. Many other hosts should be used for host range testing. Other procedures may require different hosts than the isolation host. For example, it may be found that the isolated phage reproduces more efficiently on a different host from the isolation host. And while the isolation host may be chosen because of its relationship to likely pathogenic bacterial targets, a different production host may be used because of easier purification, lack of contaminating toxins, etc. [70,71,72]. It is important to remember therefore that the requirements that guide the choice of hosts can change at each stage of a phage therapy development.

### 2.6. Post-Infection Processing

After the enrichment phase is complete, broth cultures are typically treated with chloroform to lyse any infected cells that contain intracellular phages. Direct plating methods bypass this step (see Section 2.1 above). This technique was first developed by Sechaud and Kellenberger for *E. coli* infected with T2, T4, and λ [73]. This chloroform step is common in both isolation and propagation protocols as a way of maximizing released phages although it is not clear whether it is equally effective for all phage-host systems. In addition, it has the potential to limit the isolation of some novel bacteriophage such as those that are surrounded by a lipid envelope or filamentous phages. But even among non-lipid containing, non-filamentous bacteriophages, chloroform may inactivate some phages. Ackermann notes that about 30% of tailed phages (*Caudovirales*) are sensitive to chloroform although it is not clear if this is complete inactivation or just reduced infectivity [74]. Therefore, it can be reasonable to ask, during enrichment cultures especially, whether chloroform treatment is actually necessary, except perhaps as means of biasing against chloroform-sensitive phages.

Once cell lysis has run its course, with or without chloroform treatment, uninfected and resistant bacteria along with bacterial debris are removed by centrifugation. The cleared supernatant is commonly then filtered to further remove residual bacteria and debris. As discussed above, 0.22 μ filters are typically used although 0.45 μ filters may be used instead. At this point, the phage filtrate is now ready for testing for the presence of phage.

## 3. Detection and Characterization of Phages for Phage Therapy

The basic approach to demonstrate that a phage has been isolated remains unchanged since d’Herelle began isolating novel phages. A sample of phage filtrate is mixed with a culture of susceptible bacteria and incubated. During or after incubation, the phage-cell mixture is monitored for lysis [75]. Many ways of accomplishing this have been developed depending on the type of bacteria—whether it can be grown on solid media or only in broth—and whether simple killing or more information on the infection process is needed. Most of these methods are also used when establishing host range using a multiple potential host strains. Table 1 lists the most common approaches along with some advantages and limitations.

Of these methods, plaque formation and culture lysis are the most applicable to phage therapy. Phages that had false negatives (reproduce on a host but do not form visible plaques) would probably not be useful for phage therapy due to poor productivity with the caveat that these phage detection assays are done using laboratory conditions, not in patients where phage or host growth may vary [86]. Because plaque formation requires more plates and does not automate well (or perhaps more accurately, doesn’t automate inexpensively), some groups will use it as the second test after spot testing or culture lysis [29,87]. Because those tests tend toward having false positives rather than false negatives, they can be used for a rapid first screen and only those phages that show activity need be screened by a plaque forming test [88]. As well, some groups will combine spot and plaque testing by spotting increasingly dilute phage stocks in a series of spots on the same plate. Generally, at some dilution, only a few phages are spotted so that plaques can be observed [60,87].

Plaque formation has the advantage of being usable in any lab that can culture phage. Culture lysis can also be done by simple culture and visual inspection of the growth tube. However, with the appropriate equipment, culture lysis can be scaled-up to allow testing of many phages or putative phage stocks against many host strains which is especially useful for host range testing (Section 3.5 below). Cooper and colleagues [89], for example, used a Bioscreen C analyzer (Oy Growth Curves AB Ltd., Helsinki, Finland, http://www.bioscreen.fi/bioscreencmbr.html) to monitor bacterial growth by optical density during phage culture. In this case they were selecting for phages with increased infectivity for *P. aeruginosa* by multiple rounds of phage growth with decreasing adsorption time so that only the most rapidly infecting phages could grow. The Bioscreen C analyzer can incubate two 100-well plates so 200 phage-bacteria combinations can be monitored at the same time.

Even greater scaling-up was demonstrated by Henry and colleagues [90] and Estrella and colleagues [91] who both adapted an OmniLog™ system (Biolog, Haywood, CA, USA, https://biolog.com/) to monitor phage growth by culture clearing. This device can incubate fifty 96-well plates for 4800 individual phage-bacteria combinations. It can also be used with tetrazolium dye [81] to monitor host bacterial metabolism which decreases as phage clear the culture. Using this system Henry and colleagues [90] measured the growth of novel phages infecting *Bacillus anthracis*. They were also able to determine the kinetics of the appearance of phage-resistant bacteria and demonstrated that a cocktail of their phages prevented these resistant mutants from appearing. Estrella and colleagues [91] used the same system to characterize newly isolated phages against *Staphylococcus aureus*. This system also appears to be used in Adaptive Phage Therapeutics’ (Gaithersburg, MD, USA, http://www.aphage.com/) “Host Range Quick Test” [92,93]. Using this system they are able to rapidly identify phages from their collection that can be used to treat the particular strain of a pathogen infecting in individual patient.

After the presence of phages in the lysate is demonstrated, most workers will isolate pure strains of phage through multiple rounds of plaque purification. Generally, three rounds of growth from individual plaques are considered sufficient [60]. It is possible to extract sufficient DNA for analysis directly from phage on an isolation plate [94] but these techniques are not widely used. A few workers have studied unpurified phage filtrates as naturally occurring phage cocktails (mixture of different phages) [95]. Since phage cocktails are commonly used to expand host range for phage therapy this bypassed the work needed to characterize the individual components of the cocktail. But it also meant that there was no information on the phages comprising this cocktail beyond morphology via electron microscopy of the mixtures.

### 3.1. A Note about Cocktails

Phage cocktails for phage therapy are commonly created by combining a small number of known or newly isolated phages to create a therapeutic mixture with broader host specificity [70,96]. However, some phage therapy products, such as Intesti-phage and Pyophage, from the Eliava Institute are less well-defined, based on growth and regrowth in culture for decades with the hosts changed as new pathogen strains are detected in patients [97]. Metagenomic analysis of some of the cocktails has shown them to be complex mixtures of phages from different phage families [98,99]. Villarroel and colleagues compared two samples of Pyophage produced 17 years apart and found that the overall composition stayed stable [100]. McCallin and colleagues compared Pyophage samples manufactured in Georgia (Eliava Institute) and in Russia (Microgen) and found some similar phages but also some very different components although both cocktails were intended to treat the same types of infections [101]. That there were differences identified was not completely surprising as these cocktails have been propagated independently for years although they did originally come from a common culture. Clearly propagated cocktails are more complex than assembled ones.

### 3.2. Desirable Characteristics of Phages for Phage Therapy

While one can argue that a broad biological characterization of a phage is helpful if it is to be used for phage therapy, in practice there are a limited number of characteristics that are commonly screened for:

- Ability to clear a culture of target bacteria or some other measure of phage virulence
- Obligately lytic growth or lack of lysogenic potential
- Transduction potential
- Screening for toxin genes
- Host range

Various phage scientists determine these properties in different orders as they characterize novel phages. At the recent Viruses of Microbes V meeting in Wroclaw, Poland, July 2018, in conversation, both academic and industry researchers mentioned testing for good host range first, then testing for killing ability in their particular target system (that is, not in the laboratory) and testing candidate viruses for potential temperate growth last (P. Hyman, personal observation). Others prefer to test for obligately lytic growth first. In part this may reflect the resources needed for testing the different properties, especially if whole genome sequencing is being used as a test method, as well as different end purposes [102]. Lobocka and colleagues also include a very nice summary of these properties as well as some practical considerations such as stability in storage that would inform the choices of particular phages for phage therapy [8]. Properties such as storage ability are rarely tested in most phage characterization literature but would be important in maintaining any large collection of phages [103].

### 3.3. Phage Virulence (Culture Clearing)

The ability to completely lyse a bacterial culture is the result of two properties of a phage-host system. First, the phages must have sufficient virulence and sufficient productivity that the bacteria cannot simply grow faster than the phages can kill them. Unless a very high dose of phages can be used for the initial infection, high enough to infect every cell (a killing dose), it will be necessary for phage reproduction to increase the number of phages to the point that all hosts are infected [104,105]. Second, bacterial mutants, resistant to phage infection or killing, must appear at a relatively low rate. Otherwise a resistant population of bacteria will quickly replace the phage sensitive population and could continue the infection of the patient [106]. While in some cases such as the examples cited in Section 2.4 of bacteriophage resistant strains of *Y. pestis* and *K. pneumoniae* that became less virulent, this may not always be the case. Lenski found considerable variation in fitness of *E. coli* strains resistant to bacteriophage T4 [107]. It is also not clear how significant the appearance of bacteriophage resistant strains (also called bacteriophage-insensitive mutants (BIMs)) would be during phage therapy treatments or if they would appear at the same rate as in the laboratory where fitness differences may be less important. Atterbury and colleagues [108], when testing novel phages to modify the *Salmonella* serotype distribution in broiler chickens found that, while phage-resistant bacteria appeared after a round of treatment, they were only a fraction of the population of the target strain. This is similar to results with *Vibrio cholerae* phage [109], phages infecting *P. aeruginosa* [110], and phages infecting staphylococcal biofilms [111], all of which saw reduced fitness in BIMs relative to the phage sensitive bacteria. Also see [112] for additional examples and a broader discussion of the potential for BIMs to interfere with long-term use of phage therapy.

One caveat to equating culture clearing to efficacy of a phage for phage therapy is that during phage therapy, treatment is generally not reliant only on the phage for bacterial killing. A number of studies have demonstrated utility in combining phage and antibiotic therapies [113,114]. As well, most patients will have a functioning immune system so that reducing the number of bacteria below some threshold may be sufficient for the immune system to block or destroy the remaining infection. Several modeling studies suggest there would be efficacy of phages in biocontrol in reducing the number of pathogenic bacteria below an infectious dose [106,115,116] but there is only limited experimental data to support this [117].

A second, and perhaps more critical caveat, is that phage activity should ultimately be confirmed in the same environment in which the phage will be used for biocontrol of bacteria. Clearing a broth culture of bacteria is not equivalent to killing bacteria on the surface of an infected plant or animal [15,118,119,120,121]. Likewise, clearing a broth culture of planktonic bacteria is not equivalent to degrading a biofilm [122,123,124].

### 3.4. Obligately Lytic Growth and Gene Screening

All known phages infecting *C. difficile* are temperate phages [40] but this appears to be an anomaly as most other pathogenic bacteria are known to have both obligately lytic and temperate phages. It is commonly accepted among those doing phage therapy that lytic phages are preferred over temperate phages for three main reasons [7,8,65,125]:

- Temperate phages can carry toxin genes that act as virulence factors for lysogenized bacteria raising the prospect of creating a more virulent pathogen in patients undergoing treatment [126,127].
- Many temperate phages are capable of genetic transduction which can carry genes from one bacterium to another with the potential of increasing virulence of the recipient bacterium. As well, some species of bacteria use transducing phages to move pathogenicity islands into strains lacking them at even higher efficiency than random transduction by including packaging signals on the pathogenicity islands [128,129,130].
- Lysogenized bacteria usually become immune to lytic infection by the lysogenizing phage and other phages that share similar repression systems [131,132].

Newly isolated phages are commonly screened first by looking at zones of lysis in spot testing, or better, in terms of plaque morphology. For most phages (DNA genomes, tailed), clear plaques are considered an indicator of a lytic phage while turbid plaques (especially those that are turbid in their centers) may indicate a temperate phage [60,133]. This is not definitive and so phages can be screened for the presence of integration/excision genes either by nucleic acid hybridization or PCR [7,134] or by whole phage genome sequencing and screening for these genes [135,136]. Phages may also be screened for toxin genes in the same ways, often at the same time as testing for genes associated with a temperate life cycle and potential for transduction [7,137]. For screening of multiple properties, whole phage genome sequencing is becoming the preferred method especially as the cost of genome sequencing has dropped in recent years [138,139]. There are now numerous software systems for analyzing phage genomes as well as establishing phylogenies with other phages, identifying particular types of genes, and so on. A full description of these is beyond the scope of this review but interested readers should consult recent summaries [138,139], and the additional references listed in Table 2. Software to link phages to specific hosts is described in the next section.

While in most cases it is simpler to find obligately lytic phages to target any particular bacterial species, some groups are looking at the possibility of genetically modifying temperate phages (as well as lytic phages) to enhance their use in phage therapy to deliver antibiotic sensitizing genes for example. See Manoharadas and Blasi [140], Costa et al. [141], and Pires et al. [142] for recent reviews of this.

### 3.5. Host Range

Host range is a critical property for deciding the usefulness of a particular bacteriophage for phage therapy. [In this section and beyond, host range is synonymous with productive host range as defined by Hyman and Abedon [143], that is, bacteria which are able to support phage infections which produce new phage virions.] As mentioned earlier, the greater the breadth of host range within the target pathogen species (that is, how many different strains are infected), the more likely a particular phage can be used for any particular infection by that target pathogen. Ideally, a phage will not infect other species, both because this may kill nonpathogenic members of the normal flora and because it may dilute the effective concentration of the phage toward the target bacteria although the situation will be more complex if the infection of non-target bacteria are productive infections. While host range is sometimes thought of as a matter of the correct receptor being present on the target bacteria, additional constraints on host range include bacterial anti-phage defenses such as CRISPR, restriction enzymes, and toxin-antitoxin systems (see host range reviews [143,144]. Phages have countering systems as well so that host range is a dynamic property that can change over time [145,146].

There are several ways commonly used to measure host range, some being more accurate and useful than others. I have already discussed the basic procedures in Table 1 in the context of detecting newly isolated phages. For host range testing, the chosen procedure is repeated with each host species/strain in combination with each phage being tested. As I will describe in the next section, some workers will test hundreds of strains, so a method that can be automated or that uses only small amounts of media is more economically desirable. As long as one does not rely on spot testing alone, the other methods shown in Table 1, by themselves or in combination, are sufficient to accurately define host range with one important caveat. Of necessity, host range is measured in the laboratory and target bacteria interactions with phage may be altered in vivo changing the effective host range and leading to a failure of phage therapy tests [15,118,147].

One potential new way of linking phages to their hosts is the use of genomic sequences to predict host ranges [148,149,150]. While accuracies of 60–80% are insufficient to replace in vivo testing, this is a promising area that will likely become more important as the software tools and associated databases are improved.

How many strains must be screened is in part a function of the diversity of the target species. Some bacteria such as *E. coli* and *Salmonella* are highly diverse both in surface molecules and phage susceptibility [151,152,153]. In contrast *B. anthracis, Y. pestis* and drug-resistant *S. aureus* are less genetically heterogeneous [154,155] and so correspondingly lower number of hosts may need to be tested. I will return to the subject of the number of strains that must be tested in the final section of this paper.

### 3.6. Other Properties

While not essential, other properties are often determined as part of the characterization of phages that will be used in phage therapy. Bull and Gill [156] suggest that some of these may be more predictive of success of a phage in phage therapy by allowing the building of a population dynamics model. These can also help especially in choosing components of a phage cocktail in which phages of different host ranges are mixed to create a single therapeutic agent with much broader host range than any of its components. A cocktail of phages should be less prone to lead to resistant bacteria as well [7,8]. Table 2 lists the additional properties that are more frequently tested.

In peer-reviewing several manuscripts recently, I have noticed that some researchers are determining another “property” that actually is not an intrinsic characteristic of any phages. This “property” is also appearing in published papers [171,172,175,176]. The property is multiplicity of infection (MOI). MOI is defined as the ratio of the number of phage infecting a number of bacterial host cells. If the efficiency of infection is 100% and the number of phages is much less than the number of cells, then the MOI is simply determined from the titer of the phage and bacteria at infection. When the number of phages added approaches or exceeds the number of cells, the number of cells infected will not be directly proportional to the number of phages but will follow a Poisson distribution of uninfected, singly and multiply infected cells [60] so that the MOI is an average of cells infected by different numbers of phages. The effective MOI can further change if the efficiency of infection is not 100% (which may very well what would be seen during phage therapy treatment). Efficiency of infection, and thus effective MOI, can be changed by changing environmental conditions [60]. MOI is, therefore, not a characteristic of the phage-host combination alone. More specifically, determining the optimal MOI for producing the highest titer phage stock or for cell killing in broth is not informative of the optimal MOI for treatment in phage therapy or for other procedures. For a more thorough discussion of uses of MOI measurements in phage therapy see Abedon [177].

## 4. Current Practices in Host Range Testing

Most of the above is based on the published literature which is of great value to scientists, but in preparing this article I decided to also reach out to phage scientists to determine their ideas on how phages isolated for phage therapy or related uses should be characterized for host range. While the literature is excellent at communicating what was done in a particular study, it is less effective in conveying what is being done day-to-day that may or may not ever be published. As well, questions such as the importance of host range testing are not usually noted in published work. This outreach was done in two ways. I distributed a survey at the 2017 Evergreen International Phage Meeting and later sent the same survey to the Phage-list list-serv (Phage-List@imperial.ac.uk). A total of 52 people returned the survey although not everyone answered all questions. I also spoke with several people who work in phage-related companies who felt comfortable talking in general about their practices while asking not to be quoted directly.

In the surveys, there was strong agreement (50 of 52) that host range testing is important to characterize novel bacteriophages. Most respondents (40 vs. eight) felt that spot testing alone was insufficient for characterizing host range. Several respondents did point out that, as noted above, spot testing can be used as a first test but needed to be followed up by more accurate testing such as plaque formation. In fact 65% of the respondents to the question on the method(s) they used chose spot and plaque testing. A third question asked about the types of bacterial strains used for host range testing. A minority of respondents (five of 50) felt that well-established laboratory strains alone were adequate while the majority indicated novel environmentally or clinically isolated host strains were needed.

**Figure 1:**
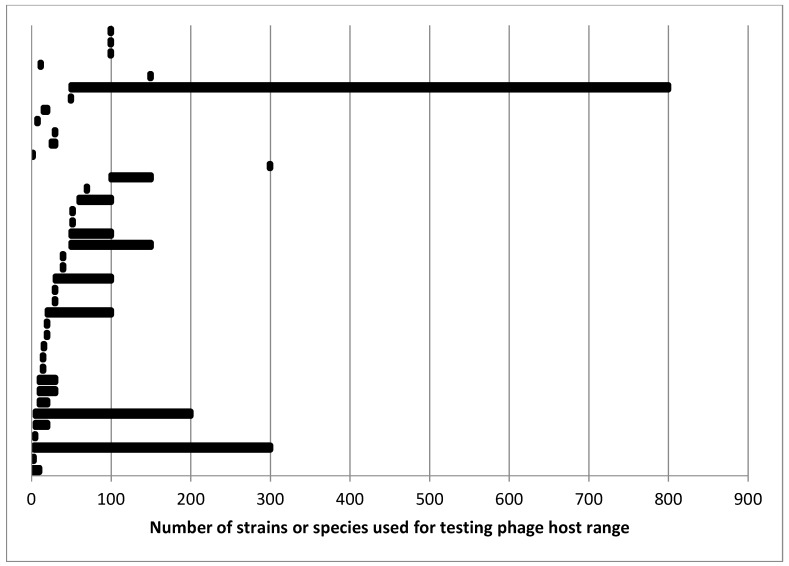
Responses to the question “How many strains do you actually use in testing host range?” The Y-axis is valueless with each horizontal line or point representing an individual response to the question.

More diversity of opinion came in response to the question “What is the minimum number of strains/species needed to adequately describe host range as narrow or broad?” About a fifth of the respondents refused to give a number writing that it depended on the host, the purpose of the characterization, and so on. Several indicated that it is not the number but the diversity of hosts that matters. All of this is true of course. For the people who did supply a number or a range of numbers, there was very little agreement. Figure 1 summarizes the responses to a related question on the number of strains they actually use for typical host range determination. Some people had a relatively small range or even a single number while others reported a range of number of strains needed with comments indicating that it depended on the type of bacteria—either by species or by group suggesting differing opinions on the diversity of bacteria and/or bacteriophages.

Several comments either in the surveys or in conversations are worth noting. Several people made the point discussed earlier on the potential for host range to be different outside of laboratory conditions. One person described host range testing when one has a large and growing host collection as an on-going process. As new hosts are acquired, phages showing some promise for whatever purpose can be tested against them. One interesting observation by another person was that host diversity could be considered in terms of where the host was isolated. For development of a phage or phage cocktail that will be distributed to distant locations, the hosts should be isolated from an equally large area in case there are geographically distinct subpopulations of hosts causing what is ostensibly the same disease. This idea is analogous to the development of antibiotics which may also be tested against a wide range of target strains and species especially using clinical strains from regions where the infection is endemic [178,179]. FDA guidance on antibiotic testing [178] may also be informative as to the number of strains that should be tested for host range. For antibiotics, the guidance is to test at least 100, and for some species, more than 300 strains and to include recent clinical isolates as at least 75% of the strains ([178], Appendix A). This is within the range that survey takers reported for numbers of strains tested for host range although above most of the responses (Figure 1).

One last question from the survey asked about the types of hosts used in more detail than just laboratory strains vs. environmental or clinical isolates. Table 3 shows the responses to this question. While only a few people tested distantly related species, there were roughly equal responses of “strains of one species”, “closely related species”, and “as diverse as possible”. There are, of course, practical considerations of time and resources that may affect this answer as well as the previous question of how many hosts to check.

## 5. Conclusions

Most phages, especially those intended for phage therapy, are isolated using slight variations on the classic enrichment protocol. A subset of phages are isolated using a mixture of hosts with the intent of obtaining broader host range phages and several protocols have been developed specifically to select phages with increased host range or polyvalent phages. These latter protocols are not in wide use currently. Instead, host range is characterized after isolation and a diversity of host ranges are seen. While most groups use methods that rely on phage growth to test host range, there are still some who rely on less accurate, but easily performed, spot testing alone.

One of the challenges with post-isolation characterization of host range is the number of hosts to be tested. As suggested by Figure 1, opinion on this varies fairly dramatically among phage workers. In part this is a function of resources both in material and time. Maintaining a collection of hosts composed of hundreds or thousands of strains and testing each new phage against even a subset of these is labor-intensive unless one has access to high-throughput automated methods which have their own resource requirements.

For those seeking FDA or other government agency approval of a phage therapy product, the question of how many strains to test is important both from a scientific viewpoint but also from a regulatory viewpoint. While there is no formal guidance yet, the example of the FDA guidance for antibiotic testing on multiple bacterial strains and species may be instructive. This suggests that hundreds of bacterial strains need to be tested for sensitivity to each newly isolated phage. It also suggests that the strains used, to the extent possible, should mirror the strains infecting patients, which means using clinical isolates and considering the location in which hosts were isolated as well as their identity. This is more extensive testing than is sometimes done when initially characterizing a phage’s host range but it should increase the likelihood that a phage therapy product will be successful in treating many cases of the target pathogen infection.

## Figures and Tables

**Table 1:** Methods for detecting newly isolated bacteriophages.

Method	Description	Advantages	Limitations *	Example References
Spot testing	A plate is inoculated with host bacteria to form a lawn, then small drops of phage filtrate are placed on the surface. After incubation a zone of lysis indicates presence of phage.	Simple. Allows testing of multiple phage filtrates on the same plate.	Host must grow to confluence on solid media. Prone to false positives due to bacterial killing by media components or phage binding that does not lead to a productive infection.	[76,77]
Plaque testing	Increasing dilutions of phage filtrate are mixed with bacteria and placed on plate surface by spreading or soft agar overlay. After incubation plate is checked for the appearance of plaques.	Demonstrates productive phage growth. Plaque appearance can suggest lytic vs. temperate life cycle. Plaque size may suggest phage size due to diffusion effects (larger phages diffuse more slowly, etc.)	Host must grow to confluence on solid media. Not all phages are capable of forming plaques even on productive hosts due to limited diffusion in agar or low productivity.	[78,79,80]
Culture lysis	Phage filtrate added to broth culture of bacteria and incubated. Monitored for cell lysis as indicated by loss of culture turbidity. Metabolic dyes can be used instead of turbidity to assess bacterial metabolic activity [81].	Useful for bacteria that will not grow to confluence on solid media as well as any bacteria that grows well in broth. Can be adapted to automation using spectrophotometry to measure turbidity either in single tube or multiple well plates.	As with spot testing, can have false positives due to non-productive lysis. Cell debris from cells lysed during early infections may bind to and inactivate free phages, interfering with later infections. Hosts that rapidly evolve phage resistant mutants will cause false negatives as mutants maintain turbidity.	[25,82]
Routine test dilution (RTD)	Phage lysates are diluted to the point of producing just less than confluent lysis on a plate.	Useful with phage that do not form distinct plaques or very small indistinct plaques.	Prone to false positives when media components are not highly diluted.	[83,84,85] †

* These limitations can also be viewed as a screening method when isolating phages for phage therapy. That is, phages that cannot form plaques or whose host rapidly evolves resistance may be poor candidates as therapeutic phages. † All of these references used RTD for host range and other characterization, not detection of newly isolated phages, but in principle RTD could be used with a high titer, poorly plaquing novel phage.

**Table 2:** Properties that are often tested in characterizing phages but are not essential for phage therapy.

Property	Description (Reference for Methodology)	References (Examples)
Efficiency of plating (EOP)	Number of plaques or lysis measurements are compared to a reference phage/host combination for relative EOP. Number of plaques compared to the number of phage particles (as determined by a non-culture method such as epifluoresence microscopy counting) used for infection is the absolute EOP. EOP between any phage pair may vary on different hosts. Often performed when multiple phages have been isolated to establish which are more or less virulent, especially when planning to combine phages to make a cocktail. EOP of each cocktail component prevents use of phages with very low killing efficiency [60].	[157]
Phage morphology by electron microscopy	Until the advent of next-generation sequencing of phage genomes, morphology was considered essential for classifying novel phages and phages of different morphologies were often used to make a broader, more diverse phage cocktail. Classification is now more commonly done by genome comparisons but knowing the morphology is still useful for quickly and easily showing diversity in a phage mixture [158].	[159,160]
Whole genome sequence	If not determined earlier, this can confirm lack of toxin genes and ability to form a lysogen. Also somewhat useful for identifying related phages for inferences of some gene functions, for example [135]. For overviews of sequencing methods see [62,135,161,162,163,164]. Phage taxonomic classification is still under development so using sequence data for this is potentially limited [165,166].	[167,168]
One step growth curve	Phages with long latent periods may be less useful as therapeutic phages. Burst size can also be determined if the treatment will be with fewer phage than can immediately infect all the bacteria infecting the patient. As discussed elsewhere in this paper, it is also important to remember that growth rates in the laboratory may not reflect growth rates in patients, in biofilms, and in other potential phage application locations [60,169,170].	[171,172]
Pulse-field gel electrophoresis (PFGE)	PFGE is a modification of conventional agarose gel electrophoresis which uses a variable electric voltage to drive DNA migration instead of the standard continuous voltage. This allows for the separation of much larger pieces of DNA. It can be used to directly measure the size of a phage genome. It can also be used for restriction fragment length polymorphism (RFLP) analysis when multiple phages have been isolated to show that each phage is different by comparing restriction enzyme digested genomic DNA although depending on the size of the phage genome, this differentiation can also be done using conventional gel electrophoresis. [173]	[174,175]

**Table 3:** Responses to question on the types of hosts to be used for host range testing.

Multiple strains of one species	14 (29%)
Closely related species	13 (27%)
Distantly related species	4(8%)
As diverse as possible	11(22%)
Multiple answers	7 (14%)
